# Te-containing carbon dots for fluorescence imaging of superoxide anion in mice during acute strenuous exercise or emotional changes†
†Electronic supplementary information (ESI) available. See DOI: 10.1039/c7sc03878j


**DOI:** 10.1039/c7sc03878j

**Published:** 2017-11-08

**Authors:** Wei Zhang, Ruixia Wang, Wei Liu, Xin Wang, Ping Li, Wen Zhang, Hui Wang, Bo Tang

**Affiliations:** a College of Chemistry , Chemical Engineering and Materials Science , Collaborative Innovation Center of Functionalized Probes for Chemical Imaging in Universities of Shandong , Key Laboratory of Molecular and Nano Probes , Ministry of Education , Institute of Biomedical Sciences , Shandong Normal University , Jinan 250014 , P. R. China . Email: tangb@sdnu.edu.cn

## Abstract

Acute strenuous exercise and emotional changes are closely related to important aspects of human health. The superoxide anion (O_2_˙^–^), as one of the primary reactive oxygen species (ROS), is intimately associated with major diseases. However, there is no relevant *in vivo* research for directly indicating the link between O_2_˙^–^ level and acute physical exercise or emotional changes. Hence, we constructed three fluorescent probes for the detection of O_2_˙^–^, including a Te-containing molecular probe and Se- and Te-containing CDs, and evaluated their properties such as selectivity, sensitivity, instantaneity and dynamic response to O_2_˙^–^. Through performance comparisons, we found that the Te-containing CDs exhibited reversibility, instantaneity and the highest sensitivity (LOD ∼ 8.0 pM), under guarantees of specific recognition of O_2_˙^–^, which ensure they are suitable for tracing native level changes in O_2_˙^–^ within living systems. The probe was applied for monitoring the levels of O_2_˙^–^ in mice under the state of intense exercise, irritability and mild depression, which led to the levels of O_2_˙^–^ significantly increasing compared to the normal condition. Furthermore, we used the Te-containing CDs for real-time and dynamic imaging of O_2_˙^–^ fluxes in the brain of mild depression mice and witnessed a positive correlation between O_2_˙^–^ levels and depression. This work provides a new strategy for studying the relationship between acute exercise or emotional changes and diseases at the level of ROS.

## Abstract 2

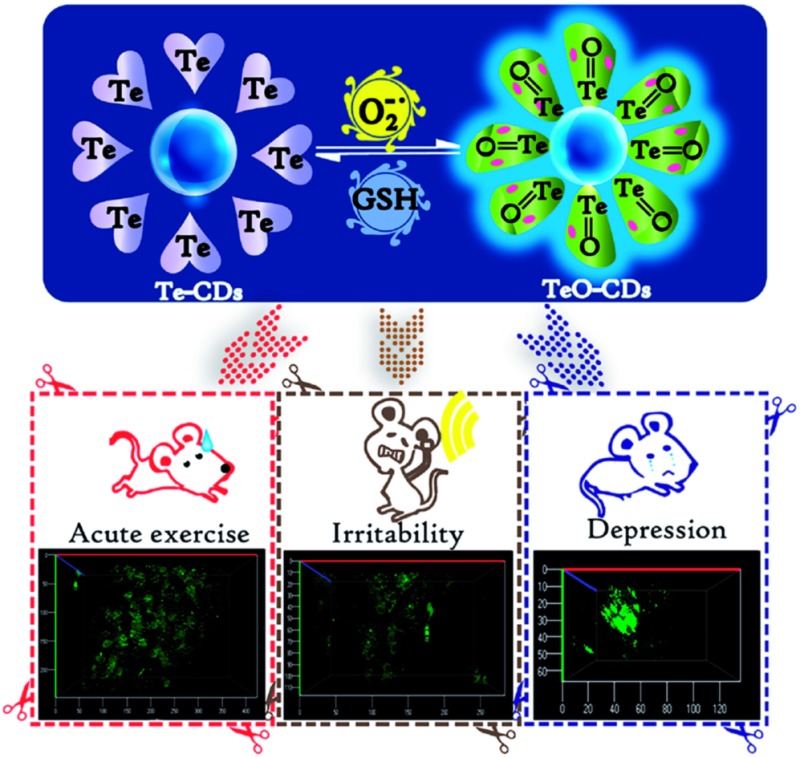
Three fluorescent probes including a Te-based molecular probe and Se- and Te-CDs were constructed and used for monitoring O_2_˙^–^.

## Introduction

Strenuous physical exercise and acute emotional changes are closely related to human health.^1^–^6^ The superoxide anion (O_2_˙^–^), as one of the primary ROS and an important signal molecule, is associated with major diseases.^7^–^9^ So are the levels of ROS, especially the first produced O_2_˙^–^, related to the state of acute exercise or emotional alterations? To explore the relationship between O_2_˙^–^ and the above-mentioned states, the fluorescence imaging method is an ideal approach because of the advantages of being nondestructive and the ability to afford high spatial-temporal resolution.^10^,^11^ Given the special properties of O_2_˙^–^ including inordinate low levels and mutual transformation between ROS in living systems, the fluorescent probes should possess an ultra-high sensitive, reversible and instantaneous response to O_2_˙^–^. Currently, with the aim to monitor O_2_˙^–^ levels in cells and *in vivo*, many analytical methods have been developed.^12^,^13^ However, limited by sensitivity and reversibility, existing imaging results of O_2_˙^–^ in cells and *in vivo* are basically achieved by external stimuli, which cannot realize real-time analysis of native O_2_˙^–^ fluctuation in biological processes.^14^–^17^ In our previous work, we developed a two-photon fluorescent probe (TCA) for dynamic and reversible imaging of O_2_˙^–^.^16^ Nevertheless, due to the detection limit of TCA being at the nanomolar level, the O_2_˙^–^ level was measured under external stimuli. In order to break through the limitation of the sensitivity of existing probes and to achieve detection of the true endogenous O_2_˙^–^ level *in vivo*, we constructed a polymer nanoprobe (PCLA-O_2_˙^–^) based on chemiluminescence resonance energy transfer, which demonstrates imaging of O_2_˙^–^ at the picomolar level in mice.^17^ However, because of the deficiency of reversibility, the PCLA-O_2_˙^–^ was not able to dynamically image O_2_˙^–^. Therefore, in order to directly uncover the linkage between the O_2_˙^–^ level and acute exercise or emotional changes, ideal fluorescence imaging probes for real-time visualization of O_2_˙^–^ levels *in vivo* are still scarce.

Currently, CDs have attracted extensive interest owing to their good biocompatibility, excellent two-photon properties, optical stability and slow diffusion. CD-based nanosensors have been used for sensing pH, metal ions, H_2_S, and so forth.^18^–^23^ To construct CD-based nanosensors, the procedure of engineering CD surfaces with diverse functions is generally complicated. Therefore, improved methods for building a CD-based nanosensor are still highly demanded. In previous studies, Te and Se have been successfully confirmed as active sites to mimetic glutathione peroxidase.^24^ These properties of Te and Se inspired researchers to design a series of Te- and Se-containing probes that can be applied for dynamic and reversible imaging of active small molecules such as ROS and mercaptan in cells.^25^–^33^ Considering that the recognition ability of Se- and Te-based active sites is mainly focused on ROS, in order to realize the dynamic fluorescence imaging of native O_2_˙^–^ fluctuation during intensive exercise or acute emotional changes, the introduction of Se- and Te-based active sites into CD-based nanosensors may offer a useful perspective on O_2_˙^–^ recognition.

**Scheme 1:**
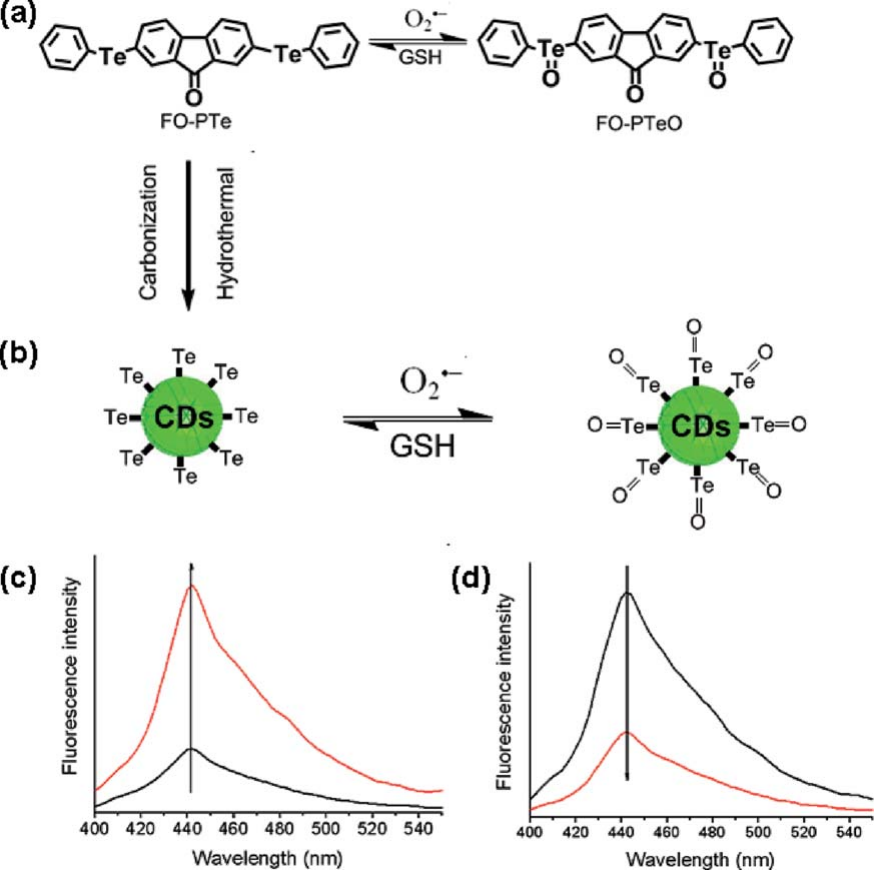
(a) Proposed reaction mechanism and structures of the molecular probes FO-PTe and its oxidized product FO-PTeO; (b) proposed reaction mechanism of Te-CD and its oxidized product TeO-CD; (c) fluorescence enhancement of the Te-CDs with O_2_˙^–^; (d) fluorescence response of the TeO-CDs with GSH.

Based on the above strategies, we developed three O_2_˙^–^ fluorescent probes (FO–PTe, Te-CDs and Se-CDs) (Scheme 1 and Fig. S1†). Among them, the Te-containing molecular probe (FO–PTe) with 9-fluorenone as a fluorophore was covalently linked with two Te-containing moieties, which could achieve dynamic and reversible detection of O_2_˙^–^ through the redox properties of the Te-center. Two other kinds of Se- and Te-containing CD were prepared from Te- and Se-containing molecular probes (FO–PTe and FO-PSe) as the carbon source, respectively. The observed results demonstrated that all three probes had good selectivity for O_2_˙^–^. More importantly, the Te-CDs and Se-CDs exhibited excellent reversibility and an instantaneous response. Their reversibility was attributed to the redox of the Te- or Se-center by further characterization. In particular, the detection limit of Te-CDs reached 8.0 pM. These probes were applied in live cells and tumor tissues to image O_2_˙^–^. The results indicated that the Te-CDs exhibited the highest sensitivity to track the endogenous O_2_˙^–^ level without external stimulation. Through two-photon fluorescence imaging, we found that the O_2_˙^–^ level significantly increased in mice during acute strenuous exercise and emotional changes. Finally, we explored the O_2_˙^–^ levels in the brains of the depressive mice using Te-CDs, which instantaneously and dynamically indicated the changes in O_2_˙^–^ concentration.

## Results and discussion

### Reaction mechanism, characterization and spectral properties of the probes

**Fig. 1:**
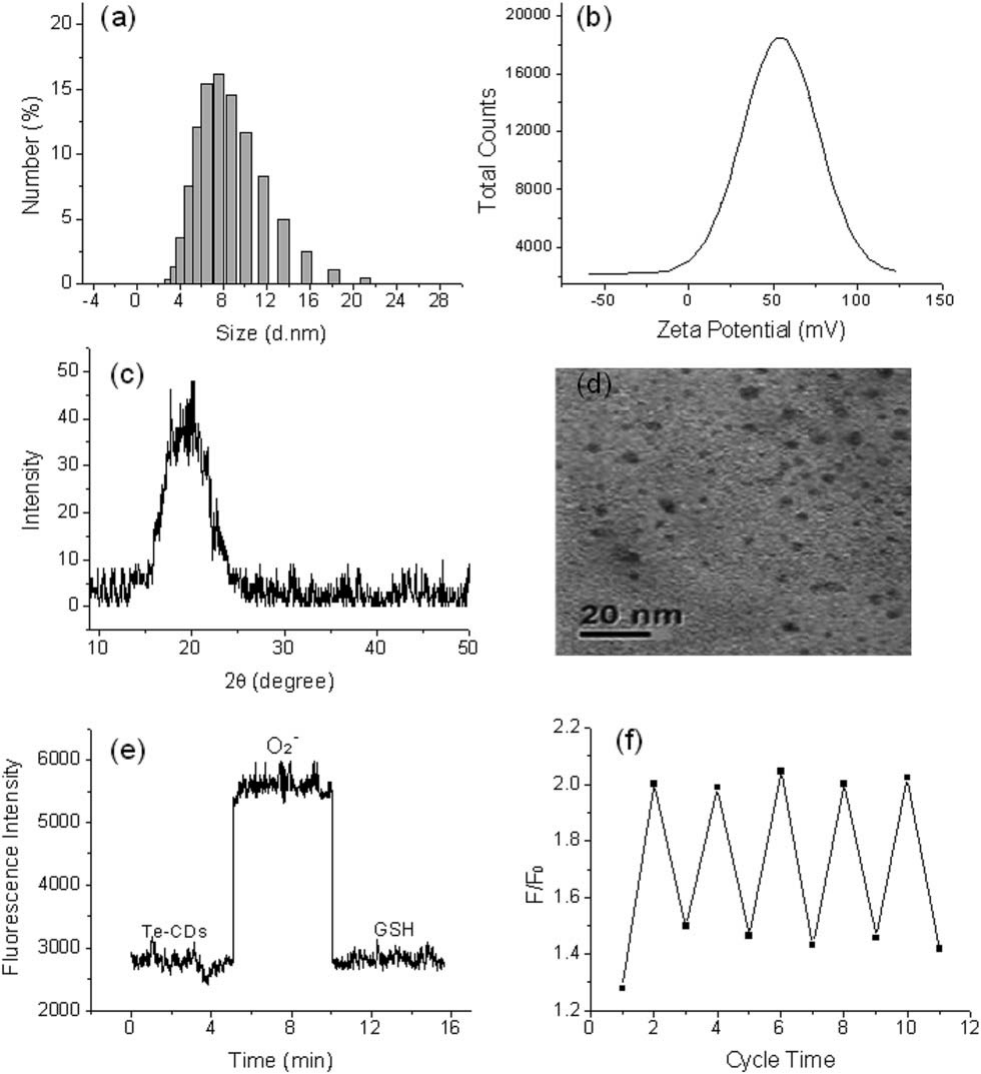
(a) Characterization of the Te-CDs by DLS images, (b) the zeta potential, (c) XRD analysis, (d) the TEM images, and (e) time course of Te-CDs (5.0 μg ml^–1^) as measured by a spectrofluorometer. The Te-CDs were oxidized by 1 equiv. of added O_2_˙^–^, after which the solution was treated with 2 equiv. of GSH; (f) fluorescence redox cycle responses of Te-CDs. The Te-CDs were oxidized by 1 equiv. of added O_2_˙^–^, after which the solution was treated with 2 equiv. of GSH.

Three fluorescent probes were constructed and the structure, reaction mechanism and spectral properties are shown in Scheme 1 and Fig. S1.† The Te-based active site with 9-fluorenone as the fluorophore was employed to fabricate the reversible organic molecular fluorescent probe FO-PTe. After reaction with O_2_˙^–^, the fluorescence intensity of FO-PTe enhanced, and the resultant product was FO-PTeO. When FO-PTeO underwent a reaction with glutathione (GSH), its fluorescence intensity became quenched. Thus, the reversible and dynamic response of O_2_˙^–^ might be realized through the redox of the Te-based active site in FO-PTe. Using FO-PTe as the carbon source, the functional Te-CDs were prepared by a single hydrothermal reaction step. The fluorescence intensity of the Te-CDs (*λ*_ex_, 380 nm; *λ*_em_, 440 nm) increased after reaction with O_2_˙^–^ (Scheme 1c), and the obtained product is TeO-CD. After reaction with GSH, the fluorescence of TeO-CDs decreased and the product is Te-CD (Scheme 1d). Through redox cycling between Te and Te=O (Scheme 1b), the probe has the ability to dynamically sense O_2_˙^–^. In order to further study the reaction mechanism and the analytical properties of the Te-CDs, we prepared Se-CDs using the molecular probe FO-PSe as a carbon source.^34^–^36^ The fluorescence response of Se-CDs (*λ*_ex_, 440 nm and *λ*_em_, 550 nm) with O_2_˙^–^/GSH was also investigated (Fig. S1c and d†). According to further characterization, we found that the structure of the directly obtained CDs was SeO-CD using the molecular probe FO-PSe as the carbon source, and then Se-CDs were produced after treatment with GSH. These results suggested that the reversible response to O_2_˙^–^ might also be realized by redox cycling of the Se-based active site. In addition, the corresponding structure and the recognition mechanism of the two CDs were well characterized by TEM, DSL, XRD, *Z*-potential, XPS and Te and Se NMR (Fig. 1a–d, S2–S7†).

### Reversibility of the probes

To determine whether the probes have reversible recognition ability, O_2_˙^–^/GSH was used to further test their fluorescence response over time. The fluorescence intensity of the Te-CDs significantly enhanced after the reaction with O_2_˙^–^ (Fig. 1e). In the meantime, the fluorescence intensity decreased rapidly when reacted with GSH, which indicated the good reversibility and instantaneity of the probe. Then the reversibility of the probe was further characterized through multiple redox cycling experiments with O_2_˙^–^/GSH (Fig. 1f). The same experiment was performed to examine the reversibility of the molecular probes FO-PTe and Se-CDs with O_2_˙^–^/GSH. The experimental results exhibited the fast reaction speed and good reversibility of FO-PTe and Se-CDs (Fig. S8 and S9†). Therefore, these results demonstrate that the redox cycling of the Se- or Te-based active site makes the probes dynamically and reversibly trace O_2_˙^–^.

### Selectivity of the probes

**Fig. 2:**
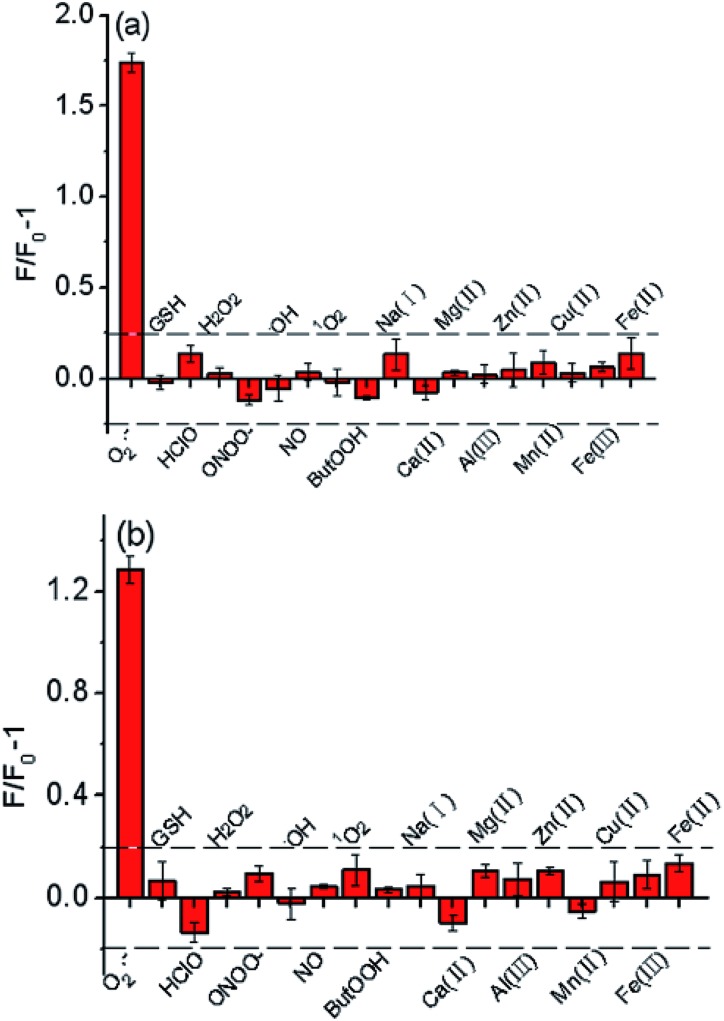
(a) Fluorescence responses of Te-CDs (5.0 μg ml^–1^) to different ROS and metal ions. The concentrations of other ROS and metal ions were 10 μM. (b) Fluorescence responses of FO-PTe (10 μM) to different active species. The concentrations of other active species were 10 μM.

To verify the selectivity of the probes, we investigated their fluorescence response to a number of biologically relevant small molecule and metal ion solutions. The fluorescence intensity of Te-CDs enhanced obviously after addition of O_2_˙^–^ (Fig. 2a), while there was little change in the fluorescence intensity in the presence of other reactive species, suggesting the great recognition ability of Te-CDs toward O_2_˙^–^. Next we confirmed the good selectivity of the molecular probe FO-PTe (Fig. 2b). As for the Se-containing CDs, we found that the fluorescence of the newly prepared CDs was significantly quenched after reaction with reducing species such as GSH and vitamin C (Vc), and the fluorescence was largely unchanged when other species were added (Fig. S10a†). After the newly prepared Se-containing CDs were treated with a reducing agent, the product fluoresced strongly when O_2_˙^–^ was added and there was low fluorescence in the presence of some other biological substances (Fig. S10b†). This confirmed that the directly prepared Se-containing CDs were SeO-CDs. Meanwhile, the structure was Se-CD after treatment with the reducing agent GSH. Altogether, these results provide strong evidence that Se or Te as active sites give the probes outstanding selectivity for O_2_˙^–^.

### Sensitivity of the probes

**Fig. 3:**
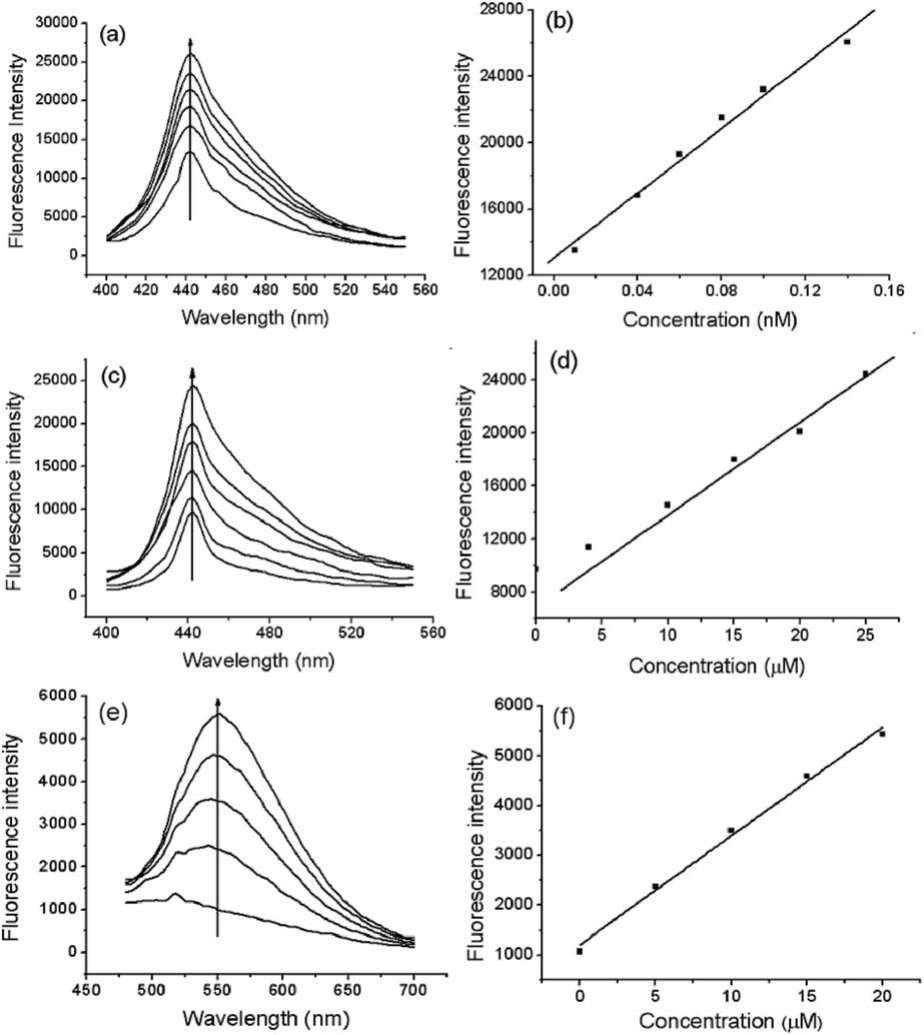
Sensitivity of the probes. (a) Fluorescence response of Te-CDs (5.0 μg ml^–1^) after adding various concentrations of O_2_˙^–^ in Tris (0.015 M) solution pH 7.4, (b) a linear correlation between the fluorescence intensity and O_2_˙^–^ concentrations, (c) fluorescence response of FO-PTe (10.0 μM) after adding various concentrations of O_2_˙^–^ in Tris (0.015 M) solution (DMSO/water = 1 : 9 v/v, pH 7.4), (d) a linear correlation between the fluorescence intensity and O_2_˙^–^ concentrations, (e) fluorescence spectra of Se-CDs (10.0 μg ml^–1^) after adding various concentrations of O_2_˙^–^ and (f) a linear correlation between the fluorescence intensity and O_2_˙^–^ concentrations.

We compared the sensitivity of the probes. Fig. 3a shows that the fluorescence intensity of Te-CDs gradually enhanced as O_2_˙^–^ concentrations increased, and the linear relationship in the concentration range of 0.01–0.14 nM had a correlation coefficient of 0.9934. Notably, the detection limit reached 8.0 pM (*n* = 11 and S/N = 3), which is a record sensitivity. Fig. 3c shows the fluorescence response of the molecular probe FO-PTe with different concentrations of O_2_˙^–^, and the linear relationship in the concentration range of 4.0–25.0 μM with a correlation coefficient and detection limit of 0.9912 and 0.15 μM, respectively (*n* = 11 and S/N = 3). Next, the response of Se-CDs with O_2_˙^–^ was also investigated (Fig. 3e). The probe showed a linear relationship in the concentration range of 5.0–20.0 μM, and the correlation coefficient and detection limit were 0.9989 and 0.12 μM, respectively (*n* = 11 and S/N = 3). By comparison, Te-CDs exhibited ultra-high sensitivity, which is suitable for the imaging of O_2_˙^–^ at native levels in cells and *in vivo* because the O_2_˙^–^ level is about 10^–10^ M in biological systems.^1^ The reasons for the difference in detection limit may be as follows: (1) comparing the Te-CDs and the molecular probe FO-PTe, there is an obviously different luminescence mechanism between the CDs and the molecular probes, and a much larger two-photon absorption cross section of Te-CDs (9707 GM, ESI†) heightening the sensitivity and (2) comparing the Se-CDs and Te-CDs, the difference of the Se- and Te-based CDs lies in the difference between Se and Te. According to the radius formula of quantum dots, the theoretical radius of Te-CDs is smaller than that of Se-CDs.^34^ Considering that the atomic radius of Te is larger than that of Se, and the specific surface area of a quantum dot increases sharply with the decrease in size, we suppose that the effective ratio of the Te-based active site in Te-CDs is higher than that of the Se-based active site in Se-CDs. The above reasons lead to the Te-CDs having the best sensitivity among these probes.

### Imaging analysis of O_2_˙^–^ in live cells

**Fig. 4:**
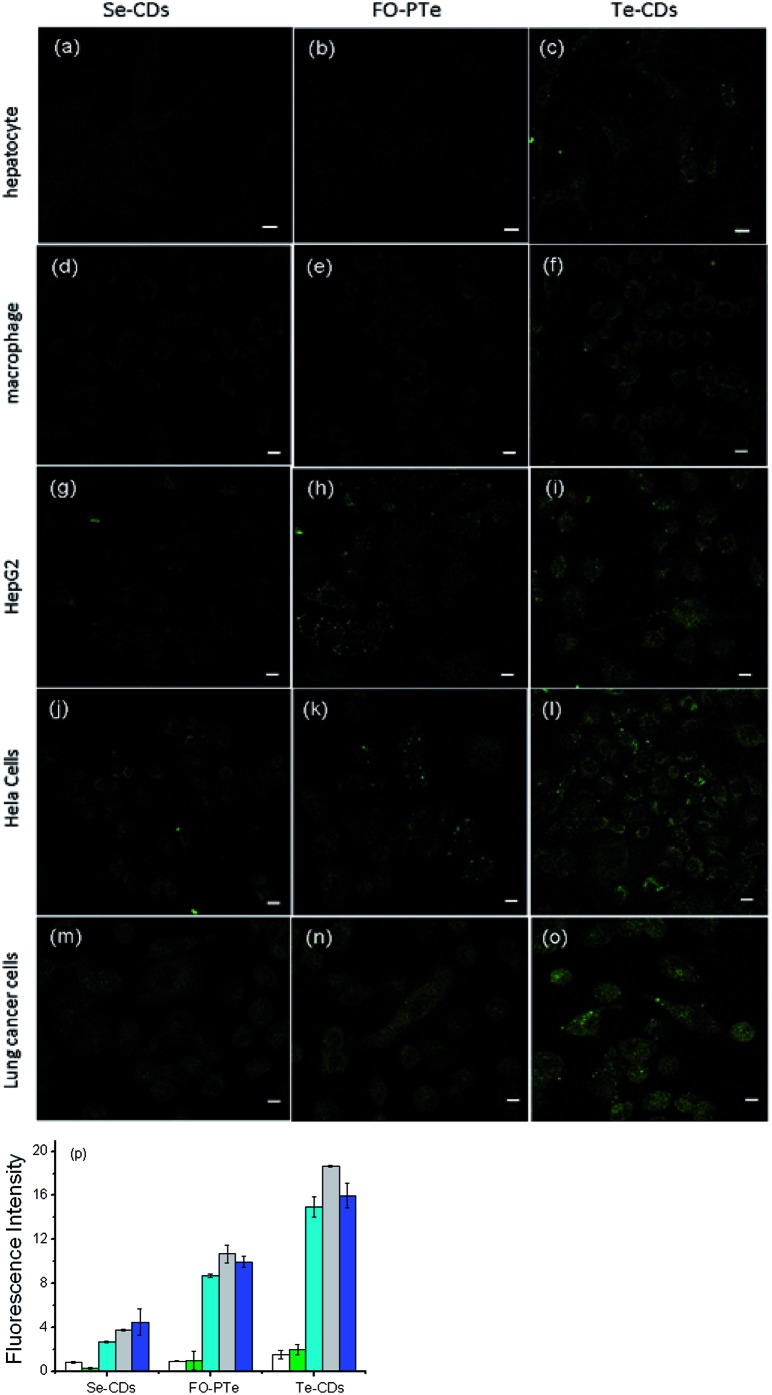
Two-photon imaging analysis of O_2_˙^–^ in cells. (a), (b) and (c) imaging of O_2_˙^–^ in hepatocyte cells by Se-CDs, FO-PTe, and Te-CDs, respectively, (d), (e) and (f) imaging of O_2_˙^–^ in macrophage cells by Se-CDs, FO-PTe, and Te-CDs, respectively, (g), (h) and (i) imaging of O_2_˙^–^ in HepG 2 cells by Se-CDs, FO-PTe, and Te-CDs, respectively, (j), (k) and (l) imaging of O_2_˙^–^ in Hela cells by Se-CDs, FO-PTe, and Te-CDs, respectively, (m), (n) and (o) imaging of O_2_˙^–^ in lung cancer cells by Se-CDs, FO-PTe, and Te-CDs, respectively and (p) the data output of fluorescence images. White: hepatocyte; green: macrophage; light blue: HepG2; gray: HeLa cells; dark blue: lung cancer cells. The concentration of the Te-CDs and Se-CDs was 5.0 μg ml^–1^ and 10.0 μg ml^–1^, respectively. The scale bar is 5 μm. Two-photon images were acquired using 800 nm excitation and two-photon fluorescence emission windows: 400–500 nm for FO-PTe and Te-CDs and 500–600 nm for Se-CDs.

We next investigated how the three probes responded to O_2_˙^–^ inside different cells (hepatocyte, HepG2 cells, macrophages, Hela cells and lung cancer cells, Fig. 4).^37^,^38^ In two-photon fluorescence images, clearer fluorescence was observed within tumor cells than normal cells, indicating higher O_2_˙^–^ concentrations than in normal cells. In particular, the Te-CDs (Fig. 4p) showed the brightest fluorescence, demonstrating a supersensitive imaging capability for O_2_˙^–^. These results manifest that the Te-CD probe possesses obvious advantages for tracking native O_2_˙^–^ in cells without external stimulation.

**Fig. 5:**
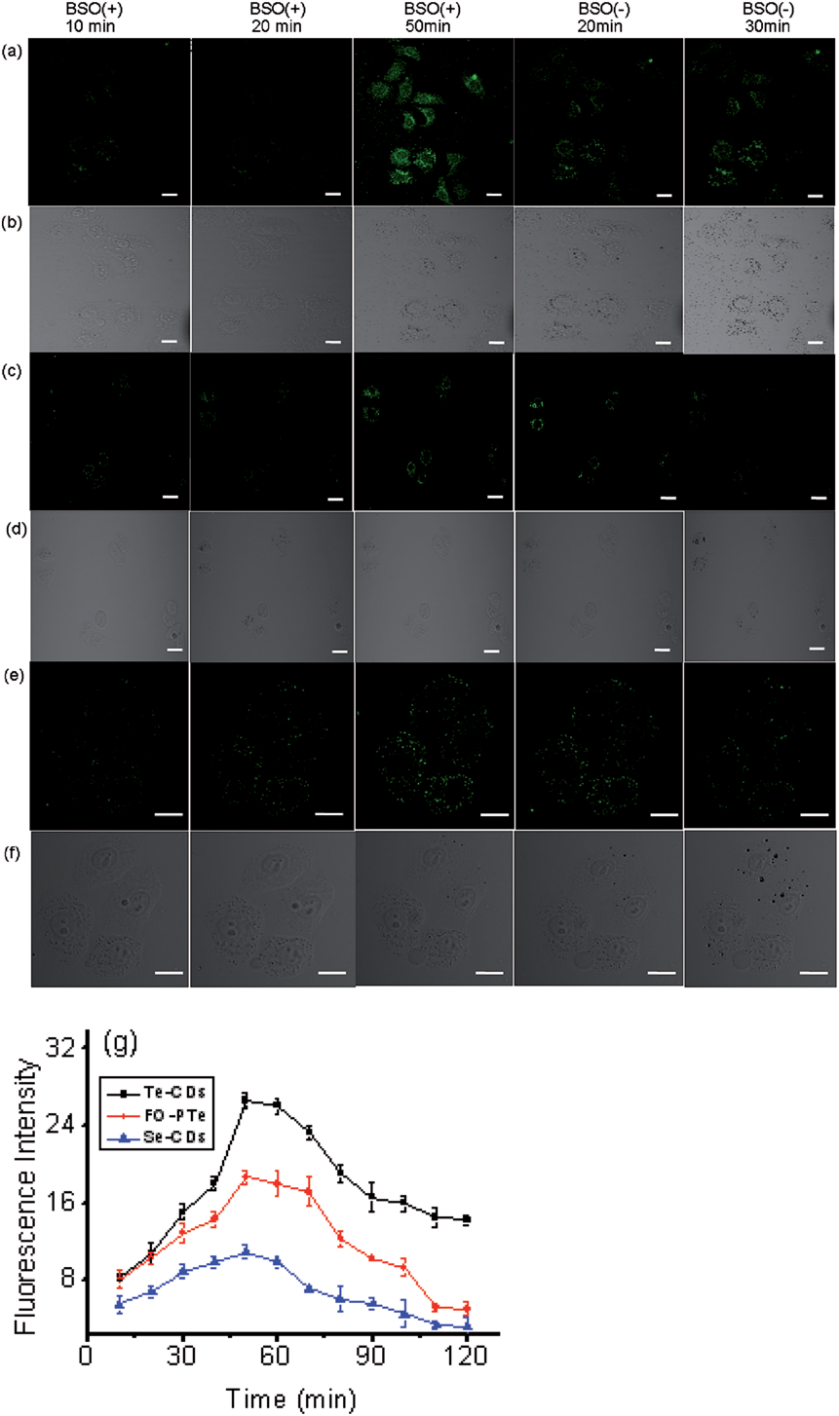
Two-photon fluorescence imaging of O_2_˙^–^ in the process of BSO-induced hepatocyte apoptosis with (a and b) Te-CDs, (c and d) FO-PTe and (e and f) Se-CDs. The CD-loaded hepatocyte cells were treated with 5.0 mM BSO. BSO was then removed, and the HL-7702 cells were further cultured in a fresh cell medium. (g) The data output of the fluorescence images. The concentration of the Te-CDs and Se-CDs was 5.0 μg ml^–1^ and 10.0 μg ml^–1^, respectively. The scale bar is 10 μm. Two-photon images were acquired using 800 nm excitation, two-photon fluorescence emission windows: 400–500 nm for FO-PTe and Te-CDs and 500–600 nm for Se-CDs.

To further test the performance of the probes for imaging O_2_˙^–^, three probes were used to monitor the changes in O_2_˙^–^ levels in hepatocytes during l-buthionine sulfoximine (BSO) induced apoptosis (Fig. 5). We observed that the fluorescence within these cells gradually elevated as time went on, which should be attributable to the rapid increase of O_2_˙^–^ during the process of apoptosis. After removal of BSO, intracellular fluorescence brightness was obviously weakened. These results suggested that these probes could measure real-time dynamic changes in O_2_˙^–^ concentrations due to their fluorescence response reversibility. Through comparing fluorescence images of the three probes, the Te-CDs also present the most distinct fluorescence, thereby proving to be equipped with fascinating characteristics for supersensitive and dynamic imaging. In addition, after addition of Tiron (superoxide scavenger), the fluorescence image brightness obviously weakened (Fig. S17 and S18†), which provides evidence that the changes in fluorescence were due to changes in O_2_˙^–^.

### Imaging analysis of O_2_˙^–^ in tumor tissue of mice

**Fig. 6:**
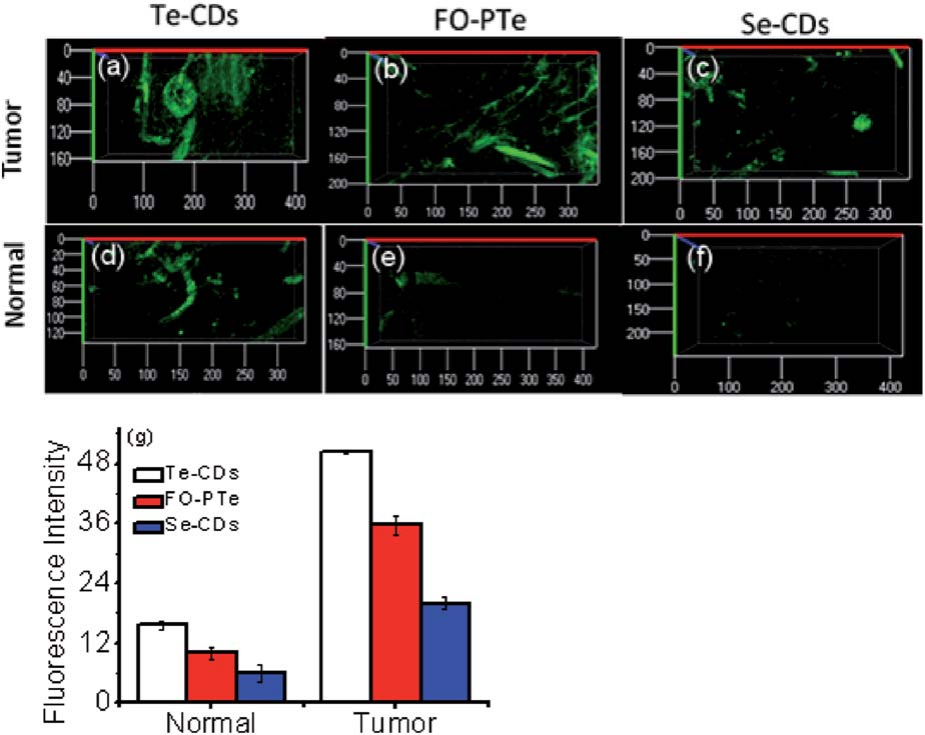
Fluorescence imaging of O_2_˙^–^ levels in the tumor tissues of mice with (a) Te-CDs, (b) FO-PTe and (c) Se-CDs and fluorescence imaging of O_2_˙^–^ levels in the normal tissues of mice with (d) Te-CDs, (e) FO-PTe and (f) Se-CDs. (g) The data output of fluorescence imaging. The error bars were calculated from three parallel experiments. The concentration of the Te-CDs and Se-CDs was 5.0 μg ml^–1^ and 10.0 μg ml^–1^, respectively. Images were acquired using 800 nm two-photon excitation, two-photon fluorescence emission windows: 400–500 nm for FO-PTe and Te-CDs and 500–600 nm for Se-CDs. Eighteen mice were used in the experiment and the error bars were from three parallel experiments.

In order to examine the feasibility of the probes in living animals, they were applied to the normal abdomen tissue and breast tumor tissue of mice (Fig. 6). Apparently, much brighter fluorescence appeared in the tumor tissue than the normal tissue. This signified a pronouncedly higher O_2_˙^–^ level in the mouse tumor tissue. These imaging results suggested that the three probes were able to track O_2_˙^–^ in small animals, combined with deep tissue penetration and reduced background fluorescence of two-photon microscopy. Significantly, the Te-CDs exhibited the best imaging quality with O_2_˙^–^, especially in the normal tissue. Moreover, the imaging depth reached more than 800 μm (Fig. S19†). This phenomenon is due to the very high sensitivity, passive targeting and slow diffusion rate of the Te-CDs. From these results, we believe that the Te-CDs have enough capability to implement the imaging of O_2_˙^–^*in vivo* without an external stimulus.

### O_2_˙^–^ fluctuations in mice under intense exercise conditions and emotional changes

**Fig. 7:**
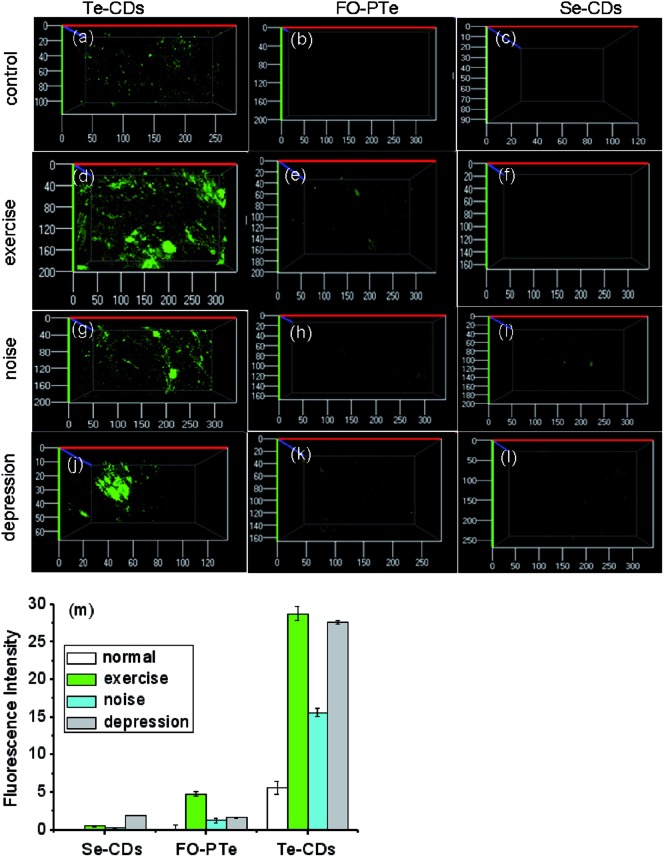
Monitoring of O_2_˙^–^ levels in states of treadmill running, noise interference and depression in mice. (a), (b) and (c) 3D distribution of O_2_˙^–^ in the normal tissues of mice (control experiment) by Te-CDs, FO-PTe and Se-CDs, respectively, (d), (e) and (f) 3D distribution of O_2_˙^–^ in the normal tissues of mice during treadmill running by Te-CDs, FO-PTe and Se-CDs, respectively, (g), (h) and (i) 3D distribution of O_2_˙^–^ in normal tissues of mice during induced irritability by Te-CDs, FO-PTe and Se-CDs, respectively, (j), (k) and (l) 3D distribution of O_2_˙^–^ in normal tissues of mice under the condition of depression by Te-CDs, FO-PTe and Se-CDs, respectively, and (m) the data output of fluorescence imaging. The concentration of the Te-CDs and Se-CDs was 5.0 μg ml^–1^ and 10.0 μg ml^–1^, respectively. The images were acquired using 800 nm two-photon excitation, two-photon fluorescence emission windows: 400–500 nm for FO-PTe and Te-CDs and 500–600 nm for Se-CDs. Thirty-six mice were used in the experiment and the error bars were from three parallel experiments.

To explore how acute strenuous exercise and emotional changes influence O_2_˙^–^ levels in small animals, we constructed three mouse models including: intense exercise of 1.5 h treadmill running, induced irritability by noise interference and mild depression after forced swimming.^39^,^40^ The three probes (FO–PTe, Te-CDs and Se-CDs) were then employed to observe O_2_˙^–^ concentrations in the abdomen of these mice (Fig. 7). Notably, the abdomen of the normal mice injected with Te-CDs displayed perceptible fluorescence (Fig. 7a), which was due to native O_2_˙^–^ levels in the control group. Furthermore, stronger fluorescence was observed for the mice under acute exercise, irritability and depressive states (Fig. 7d, g, j and m), revealing the distinct rise in O_2_˙^–^ concentrations. This means that the O_2_˙^–^ increase may lead to oxidative damage to organisms. Moreover, the superoxide scavenger (Tiron) was used to support the observed fluorescence that can be assigned to the presence and reactivity of O_2_˙^–^ towards the probe. The fluorescence image brightness obviously weakened (Fig. S22†), suggesting that the changes in fluorescence were from the changes in O_2_˙^–^.

**Fig. 8:**
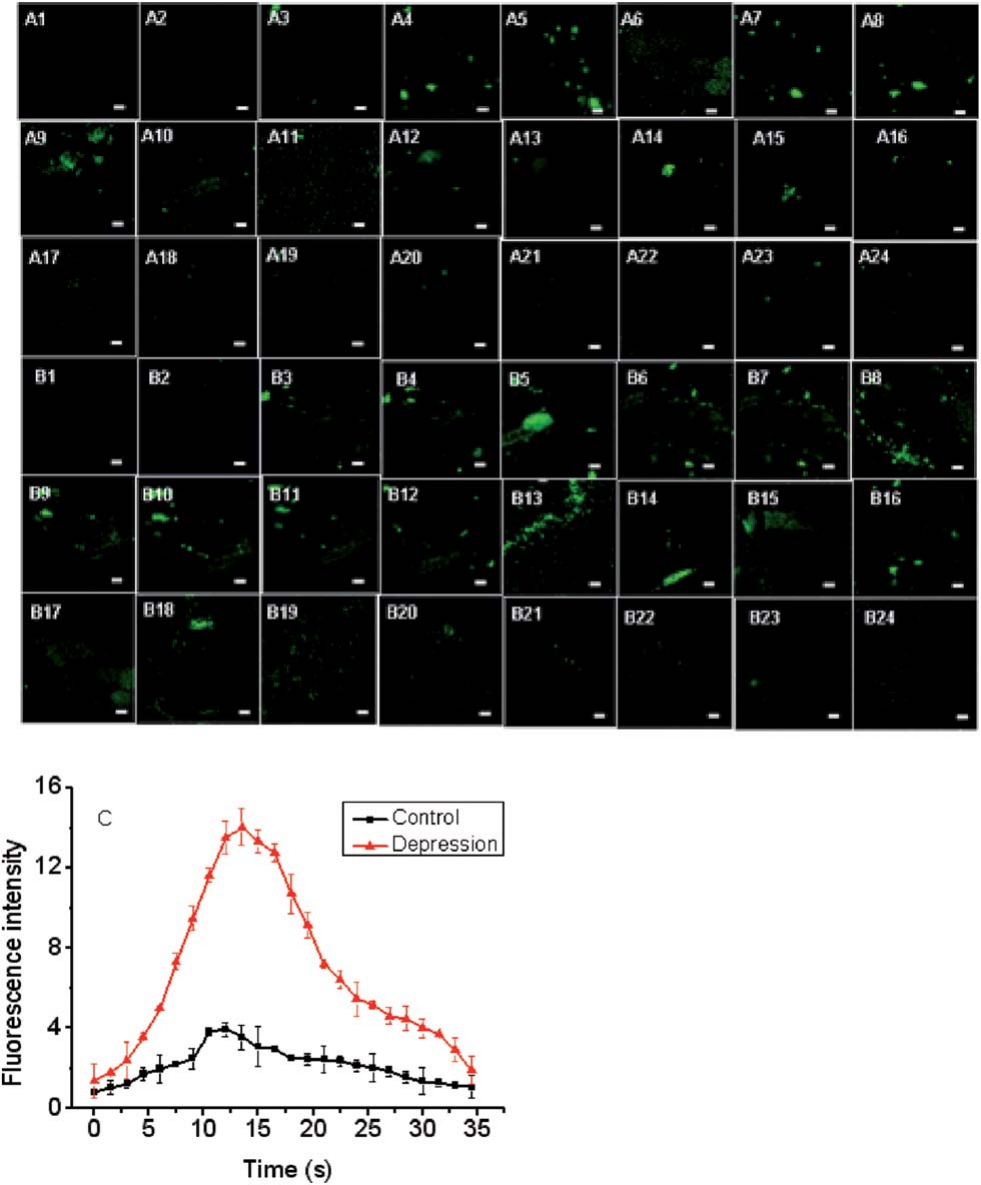
Monitoring the O_2_˙^–^ dynamic fluctuations in the brain of the depression model using Te-CDs. (A1–A24) The changes of O_2_˙^–^ dynamic fluctuations in the brain of mice, and each picture interval is 2 seconds, (B1–B24) the changes of O_2_˙^–^ dynamic fluctuations in the brain of the depression model, and each picture interval is 2 seconds; (C) data output of fluorescence imaging. The concentration of the Te-CDs and Se-CDs was 5.0 μg ml^–1^ and 10.0 μg ml^–1^, respectively. Images were acquired using 800 nm two-photon excitation, two-photon fluorescence emission windows: 400–500 nm. The scale bar is 20 μm. Five mice were used in the experiment and the error bars were from five parallel experiments.

To further confirm the changes of O_2_˙^–^ in the mild depression model, the Te-CD probe was applied in the brain for real-time imaging. We found that the fluorescence brightness increased gradually and then decreased gradually over time (Fig. 8), reflecting dynamic changes of the O_2_˙^–^ levels in the brain of the depressed mouse (Fig. 8c). We speculated that the changes of O_2_˙^–^ during imaging were mainly due to “superoxide flashes”, which involve the transient openings of mitochondrial permeability transition pore stimulating superoxide production by the electron transport chain.^15^ Compared with the control group, the concentration of O_2_˙^–^ in the brain of the depressed mice increased significantly. These results suggest a close relationship between ROS and depression.

## Conclusions

For research on health or disease, the relationship between the O_2_˙^–^ level and acute exercise and emotional changes was revealed using three probes, including Te-CDs, FO-PTe and Se-CDs, which were developed for monitoring intrinsic levels of O_2_˙^–^ based on the Se or Te active sites. These probes can instantaneously and dynamically respond to O_2_˙^–^ with specific recognition. Interestingly, Te-CDs displayed the highest and a record sensitivity with the detection limit at a picomolar level. Application of the three probes in different cells and tissues by two-photon fluorescence imaging confirmed that the Te-CDs were the most suitable for tracking O_2_˙^–^ fluctuation without external stimulation. Mouse abdomen imaging using Te-CD, uncovered that the level of O_2_˙^–^ increased sharply under intense exercise, irritability and mild depression. Furthermore, dynamic imaging in the brain of the mild depression model revealed O_2_˙^–^ at a higher level. This work provides a new strategy and ideal imaging tool to unravel the influence of acute exercise and emotional change on health from the ROS level.

## Conflicts of interest

There are no conflicts to declare.

## Supplementary Material

Supplementary information
