# Supplementary material for: Te-containing carbon dots for fluorescence imaging of superoxide anion in mice during acute strenuous exercise or emotional changes
Source: Chem Sci. 2017 Nov 8;9(3):721–7. doi: 10.1039/c7sc03878j (PMC5869990; doi:10.1039/c7sc03878j)
Supplement: Supplementary file 1 [file SC-009-C7SC03878J-s001.pdf]

Electronic Supplementary Material (ESI) for Chemical Science.
This journal is © The Royal Society of Chemistry 2017

Supporting Information

Te-containing carbon dots for fluorescence imaging of superoxide anion in mice during acute strenuous exercise or emotional changes

Wei Zhang, Ruixia Wang, Wei Liu, Xin Wang, Ping Li*, Wen Zhang, Hui Wang and Bo Tang*

College of Chemistry, Chemical Engineering and Materials Science, Collaborative Innovation Center of Functionalized Probes for Chemical Imaging in Universities of Shandong, Key Laboratory of Molecular and Nano Probes, Ministry of Education, Institute of Biomedical Sciences, Shandong Normal University, Jinan 250014, P. R. China

*To whom correspondence should be addressed. Tel.: (86) 531-86180010; Fax: (86) 531-86180017

E-mail: tangb@sdnu.edu.cn.

Materials. All chemicals were available commercially and the solvents were purified by conventional methods before use. 2,7-Dibromo-9H-fluoren-9-one, Diphenyl diselenide were purchased from sun Chemical Technology(shanghai) Co., Ltd. DL-Dithiothreitol,1,8-Diazabicyclo[5.4.0]undec-7-ene were purchased from Aladdin Industrial. 2-Methoxyestradiol and buthionine sulphoximine (BSO) from Sigma-Aldrich Reactive oxygen species were as follows. Hypochlorite (NaOCl), H_2O_2, tert-butylhydroperoxide (TBHP), and were delivered from 10%, 30% and 70% aqueous solutions respectively. Hydroxyl radical (•OH) was generated by reaction of 1 mM Fe^{2+} with 200 μM H_2O_2. Nitric oxide (NO) was used from stock solution prepared by sodium nitroprusside. Singlet oxygen ($^1O_2$) was prepared by the ClO^-/H_2O_2 system. Superoxide ($O_2^{\bullet-}$) was delivered from KO_2 in DMSO solution. Peroxynitrite was used from stock solution 10 mM in 0.3 M NaOH.

Instruments. ^{1}HNMR spectra were recorded with Bruker NMR spectrometers at 300 MHz and JOEL JNM-ECA600.The mass spectra were obtained by Bruker maXis ultra high resolution TOF MS system. The one-photon excited fluorescence spectra measurements were performed using FLS-920 Edinburgh fluorescence spectrometer. Two-photon excited fluorescence spectra were measured using a Tsunami 3941-M3-BB: Ti: sapphire femtosecond laser as exciting light source (800 nm) with a pulse width of <150 fs and a repetition rate of 80 MHz, and USB2000 (bought from Ocean Optics Inc.) was employed as the recorder. The one-photon confocal fluorescent images were measured on a Leica TCS SP5, confocal lasers canning microscope with an objective lens (×40). The excitation wavelength was 405 nm (5 mW). Two-photon confocal fluorescent images were measured on Zeiss Microsystems. UV/Vis spectra were recorded on TU-1900 UV/Vis spectrometer. X-ray photoelectron spectroscopy (XPS) measurements were performed on a Thermo Fisher Scientific Escalab 250 X-ray photoelectron spectroscopy using an Al Kα (h = 1486.6 eV) radiation excitation source. XRD pattern was obtained on D8 ADVANCE X-ray

powder diffractometer (XRPD) with Cu Kα radiation (λ = 1.5405 Å). High resolution transmission electron microscopy (HRTEM) was carried out on a JEM-2100 electron microscope. Dynamic light scattering (DLS) and Zeta potentials measurements were performed on a Malvern zeta sizer Nano-ZS90. FTIR spectra were collected on a Nicolet Impact 410 FTIR spectrometer in the range of 400-4000 cm^{-1}.

Synthesis of 2,7-bis(phenylselanyl)-9H-fluoren-9-one (FO-PTe). Diphenyl ditelluride (100 mg, 0.244 mmol) was added to a solution of sodium borohydride (50 mg, 0.8 mmol) in the mixed solution of anhydrous DMF (10.0 mL) and anhydrous alcohol (3.0 ml) under Ar atmosphere. After stirring at 115 °C for 1.5 h, 2,7-dibromo-9H-fluoren-9-one (40 mg, 0.118 mmol) was added to the reaction mixture and the mixture was stirred for 30 min. Then, 1,8-diazabicyclo[5.4.0]undec-7-ene (DBU, 0.75 mL, 5.0 mmol) was added and the mixture was stirred for further 18 h. The solvent was removed under reduced pressure and the residue was extracted with CH_2Cl_2 and saturated sodium dithionite aqueous solution. The resulting solution was dried with anhydrous $MgSO_4$. After the concentration treatment, the red brown crude product was obtained. The eluent (petroleum ether: dichloromethane =4:1) was used to isolate and purify the above-mentioned crude product. Finally get the deep orange red products. Yield: 53 mg (53%). ^{1}H NMR (400 MHz, $CDCl_3$) δ 7.623 (s, 2H; Ar H), 7.482 (d, 2H; Ar H), 7.472 (d, 2H; Ar H), 7.245−7.359 (m, 10H; Ar H). ^{13}C NMR (100 MHz, $CDCl_3$): δ 192.81, 142.59, 137.53, 133.65, 132.46, 129.09, 128.59, 127.31, 127.19, 126.69, 119.93. MS data, m/z: 587.9137 (M-H). And the molecular probe FO-PSe was synthesized according to previous literature.[S1]

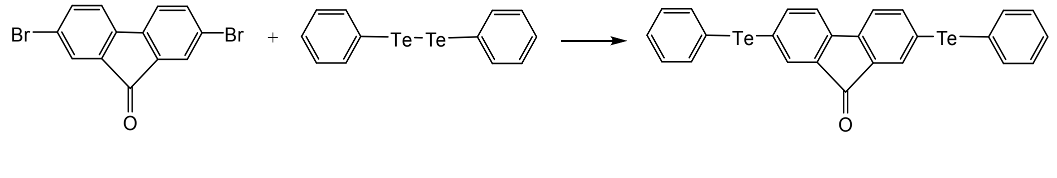

Scheme S1 Synthesis of probe FO-PTe

Synthesis of CDs. The CDs were prepared by hydrothermal treatment of FO-PTe and FO-PSe, respectively.[S1,S2] Briefly, FO-PTe or FO-PSe was dissolved in ethanol solution, and then heated to 160 °C for 12 hours. After cooling to room temperature, the resultant CDs solution was removed reduced pressure then the dialysis membrane with molecular weight of 1000 was used for purification of the CDs samples. Finally, the CDs were then dispersed in Tris solution (pH 7.4) at concentration of 0.1 mg/mL for further characterization and evaluation.

Transmission electron microscopy (TEM) and dynamic light scattering (DSL) characterizations were performed to prove the properties of the CDs. The DLS analysis showed that the CDs had an average diameter of 8 nm (Fig. 1 and S2). The surface charge of the CDs was examined by measuring the z-potential, which results was about 52 mV and indicated that the as-prepared CDs have good dispersion in water. In addition, the XRD was performed and the results were shown in Fig.1c. The as-prepared CDs showed a wide-angle XRD pattern with a broad peak corresponding to (002), which indicated the partial graphitization and small size of the as-prepared CDs (Fig. 1c and S2). The morphology and structure of the CDs were further performed. Fig. 1d showed the TEM images of the as-prepared CDs with particle diameters of approximately 8 nm and exhibit good dispersion without apparent aggregation. The Fourier transform infrared (FTIR) spectra and the X-ray photoelectron spectroscopy (XPS) survey spectra were used to further confirmed the obtaining CDs (Fig. S3, S4). In addition, the oxidation-reduction form of Te- and Se-center in the CDs was further confirmed the Te and Se NMR (Fig. S5-S7).

Cell and mice culture. The Hepatocyte cells, HepG2 cells, macrophages, Hela cells and lung cancer cells were cultured in DMEM containing 10 % fetal bovine serum, 1.0 % penicillin, and 1.0 % streptomycin at 37 °C (w/v) in a 5% CO_2/95% air incubator MCO-15AC (Sanyo,

Tokyo, Japan). The concentrations of counted cells were adjusted to 1.0×10^6 cells mL^{-1} for confocal imaging in high-glucose DMEM (4.5 g of glucose/L) supplemented with 10 % fetal bovine serum (FBS), $NaHCO_3$ (2.0 ng/L), and 1.0 % antibiotics (penicillin/streptomycin, 100 U/mL). Cultures were maintained at 37 °C under a humidified atmosphere containing 5% CO_2.[S3,S4]

Eight- to ten-week-old wild-type BalB/C mice (male) were used. The mice were anesthetized with 4 % chloral hydrate (3.0 mL/kg) by intraperitoneal injection. The mice were then imaged by using a Zeiss LSM880NLO in vivo imaging system. The mice were obtained from Shandong University Laboratory Animal Center. The experiments were approved by the institutional committee. All the animal experiments were carried out in accordance with the relevant laws and guidelines issued by the Ethical Committee of Shandong University. The breast cancer mouse model: Normal female kunming mice (about 20 g) was used for this experiment. The mouse breast cancer cells are first treated by centrifugation (6000 r/min), then diluted with saline solution. The concentration of the tumor cell suspension is 2×10^6 cells/ ml through cell count. 0.2 ml of tumor cell suspension was injected into the skin of the right axillary in experimental group mice. The tumor was formed after 7-9 days. Three weeks later, the obtained mouse model was used for the experiment. The experimental process: The probes were injected into the mouse tumor. After 15 minutes, the two-photons imaging were employed. The control group injected the same amount of saline. Each group was repeated three times.

Depression model (the forced swimming test in mice):[S5,S6] Female kunming mices (weight 20 g) were selected as the depression model for this experiment. Put mice in the cylindrical tank with 20 cm high, diameter of 14 cm, and the water temperature and depth of the water is 25 °C, 20 cm, respectively. In the forced swimming test, 15 min of swimming training was employed to make the mice produce inevitable stress and cause "depression reaction". And then feeding the mice for 24 hours, then we put the mouse in the water for 5 min and observed

the mice swimming behavior. After ten minutes, repeat the test. And then take the mouse out, injection of the CDs in the abdomen for in situ imaging. Mouse movement model: Normal female kunming mice (about 20 g) was used for this experiment. Put in the mouse cage wheel, make it keep on moving 1.5 hours. After the mice were anesthetized, the CDs-based probe was injected into the abdomen for in situ imaging. The model of mouse noise interference test: Normal female kunming mice (about 20 g) was used for this experiment. Put in the mice in the cage, and then put the cage in the ultrasonic cleaning machine for 1.5 hours and the irritability of the mice was clearly observed. After the mice were anesthetized, the CDs-based probe was injected into the abdomen for in situ imaging. Mouse brain imaging procedure: The Te-CDs (5.0 μg/ml, 20 μL) was injected into the brain of the depression model mice for in situ imaging. Fluorescence imaging of the superoxide was obtained in the depth of 20 μm. Each imaging time interval was about 1.5 s, and the imaging area was 30 × 30 μm^2. The experimental conditions of the normal group were the same as that of the experimental group. The mice were obtained from Shandong University Laboratory Animal Center. The experiments were approved by the institutional committee. All the animal experiments were carried out in accordance with the relevant laws and guidelines issued by the Ethical Committee of Shandong University.

MTT Assay. Hepatocyte cells (10^5 cell mL^{-1}) were dispersed within replicate 96-well microtiter plates to a total volume of 200 μL $well^{-1}$. Plates were maintained at 37 °C in a 5% CO_2/95% air incubator for 24 h. Then HL-7702 cells were incubated for 12 h upon different probe concentrations of 0, 0.5, 5, 50, 200, 300, and 500 μg/mL. MTT solution (5.0 mg mL^{-1}, PBS) was then added to each well. After 4 h, the remaining MTT solution was removed, and 150 μL of DMSO was added to each well to dissolve the formazan crystals. Absorbance was measured at 490 nm in a Triturus microplate reader.

Confocal imaging and two-photon fluorescence imaging. One-photon fluorescent images were acquired on a Leica TCS SP5 confocal laser-scanning microscope with an objective lens (×40). The excitation wavelength was 405 nm (5.0 mW). Following incubation, the cells were washed three times with DMEM without FBS and imaged. The two-photon imaging of cells and mouse were obtained with Zeiss LSM880NLO with a 20 × water objective All the Ti: sapphire laser was used to excite the specimen at 800 nm and transmissivity was 6.0%.

Measurement of two-photon cross section.[S7,S8] The two-photon cross section (δ) was determined by using femto second (fs) fluorescence measurement technique. The two-photon induced fluorescence intensity was measured at 800 nm by using fluorescein (1.0×10^{-5} M, pH 11.0) as the reference, whose two-photon property has been well characterized in the literature. The intensities of the two-photon induced fluorescence spectra of the reference and sample emitted at the same excitation wavelength were determined. The TPA cross section was calculated according to Eq (1).

$$\delta_s = \delta_r \frac{\Phi_r}{\Phi_s}\frac{C_r}{C_s}\frac{n_r}{n_s}\frac{F_s}{F_r} \qquad (1)$$

The subscripts s and r is the sample and the reference material, respectively. δ is the TPA cross sectional value, C is the concentration of the solution, n is there refractive index of the solution, F is two-photon excited fluorescence integral intensity and Φ is the fluorescence quantum yield. The results showed that the two-photon cross section of CDs-Se and CD-Te at pH 7.4 is 5800 GM and 9707 GM.

REFERENCES

S1 W. Zhang, W. Liu, P. Li, J. Kang, J. Wang, H. Wang and B. Tang, *Chem. Commun.,* 2015, **51**, 10150-10153.

S2 V. Georgakilas, J. A. Perman, J. Tucek and R. Zboril, *Chem. Rev.,* 2015, **115**, 4744-4822.

S3 R. Liu, L. Zhang, X. Lan, L. Li, T.T. Zhang, J.H. Sun and G. H. Du, *Neuroscience,* 2011, *10*, 408-419.

S4 S. Thiberge, S. Blazquez, P. Baldacci, O. Renaud, S. Shorte, R. Ménard and R. Amino, *Nat. Protoc.* 2007, **2**, 1811-1818.

S5 R.D. Porsolt, L. M. Pichon and M. L. Jalfre, *Nature* 1977, **266**, 730-732.

S6 H. Takeda, M.Tsuji, M. Inazu, T. Egashira, T. Matsumiya, *Eur. J. Pharmacol* 2002, **449**, 261-267.

S7 C. Xu, W. W. Webb, *J. Opt. Soc. Am. B* 1996, **13**, 481-491.

S8 J.R. Lakowicz, New York, London, Moscow, Dordrecht, 1999.

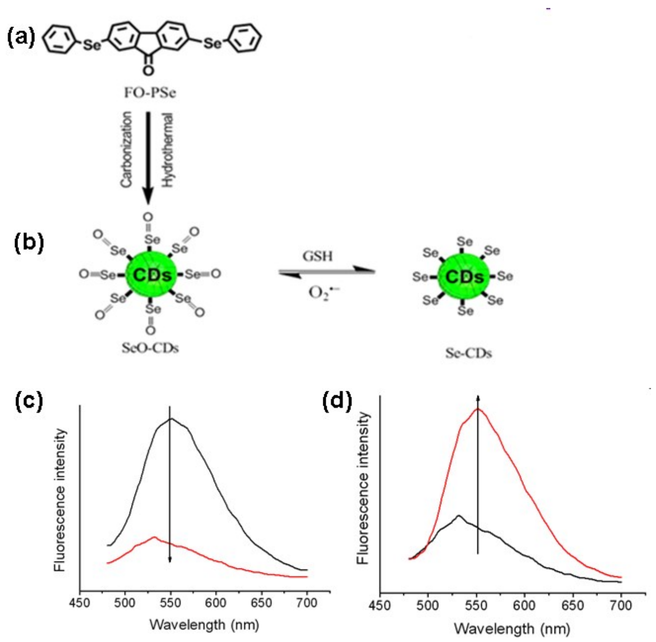

Fig. S1 (a) Structures of molecular probes FO-PSe;[S1] (b) Proposed reaction mechanism of Se-CDs and its oxidized product SeO-CDs; (c) Fluorescence quenching of Te-CDs with GSH; (d) Fluorescence enhancement of Se-CDs with $O_2^{•-}$.

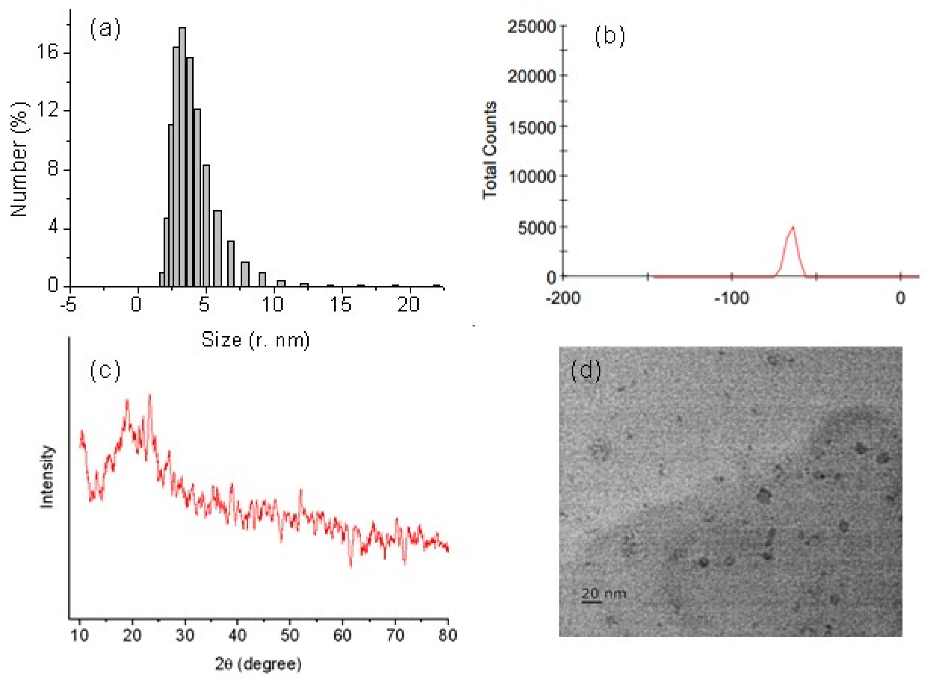

Fig. S2 Characterization of Se-containing CDs by DLS images (a), the zeta potential (b), TEM (c) and XRD (d), respectively.

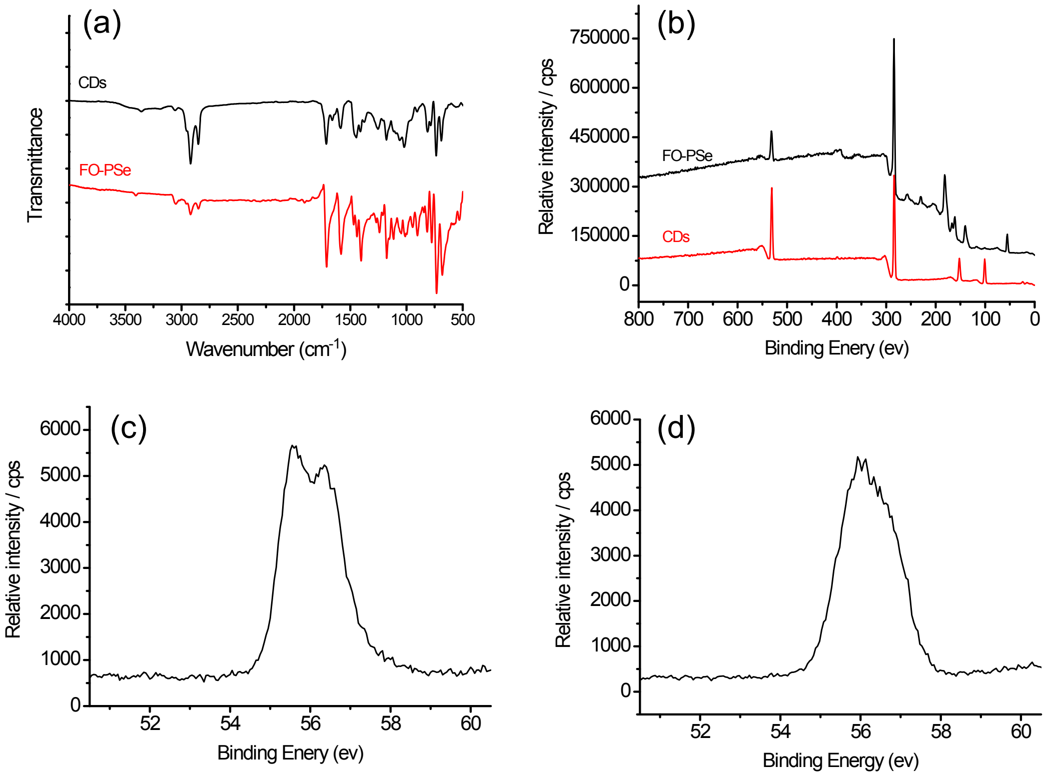

Fig. S3 FT-IR spectra of the Se-containing CDs and precursor FO-PSe (a). XPS survey (b) and fitted Se 3d of the precursor FO-PSe (c) and the as-prepared CDs (d).

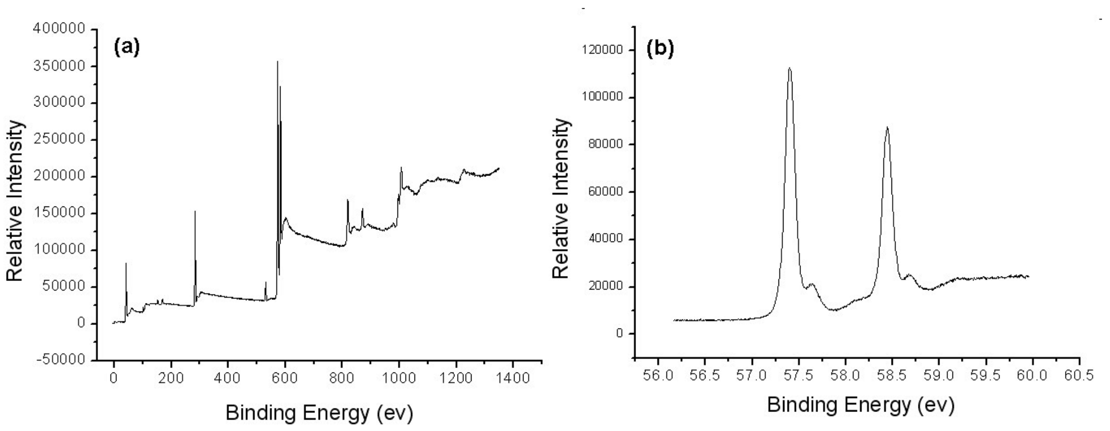

Fig. S4 XPS survey (a) and fitted Se 3d (b) spectra of the Te-Containing CDs.

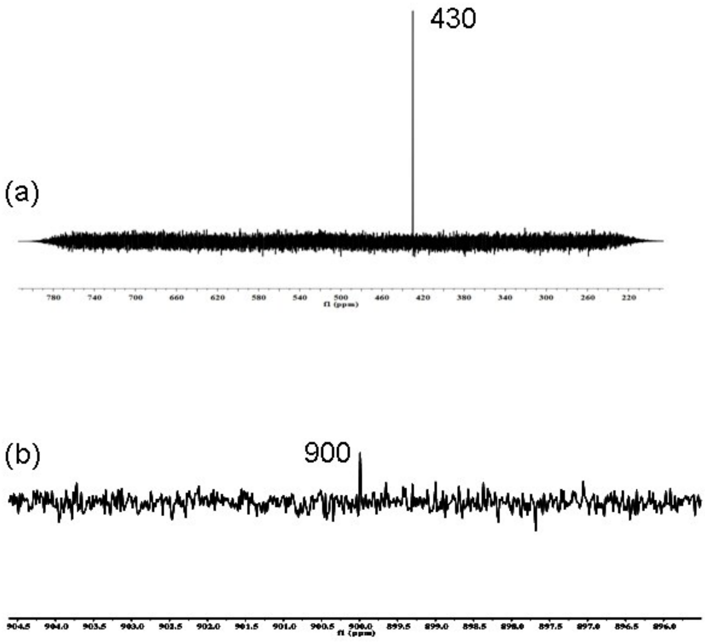

Fig. S5 Se NMR of the Se-CDs (a) and its oxidized products SeO-CDs (b).

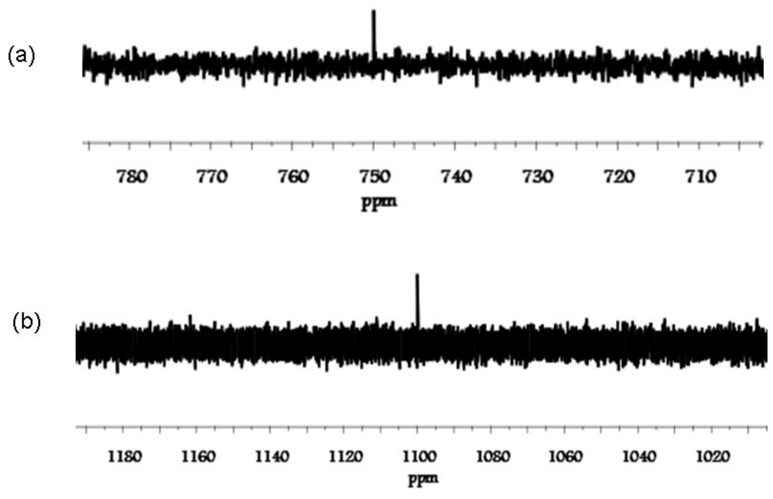

Fig. S6 Te NMR of the FO-PTe (a) and its oxidized products FO-PTeO (b).

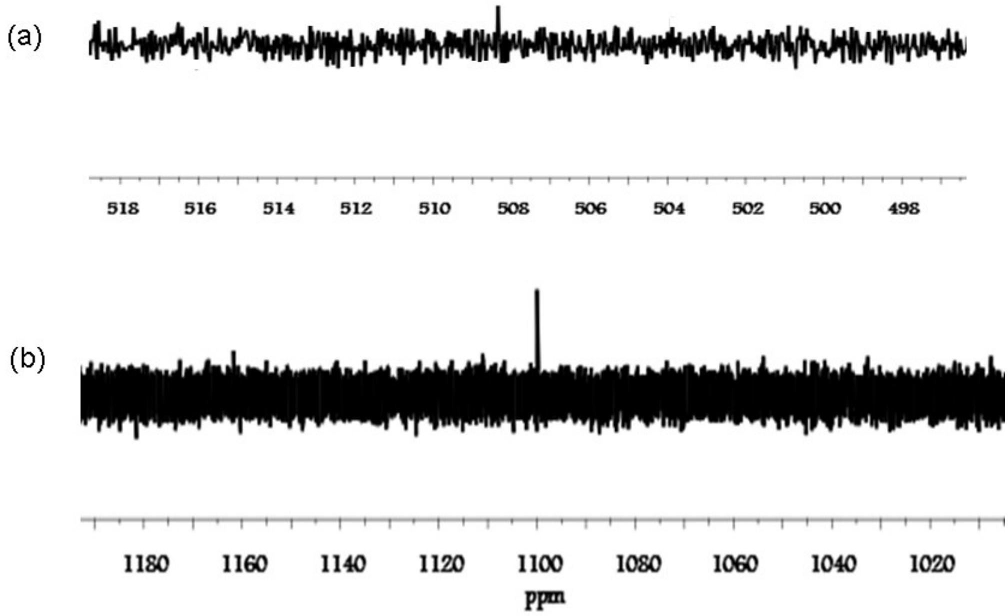

Fig. S7 Te NMR of the Te-CDs (a) and its oxidized products TeO-CDs (b).

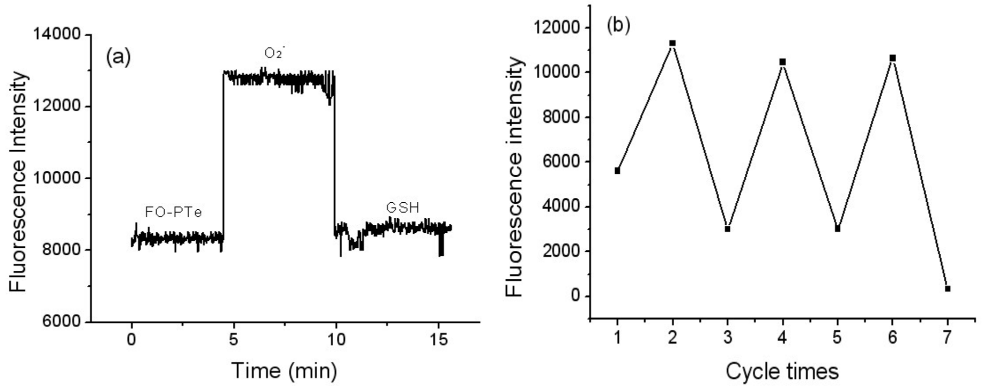

Fig. S8 Time course of FO-PTe (10.0 μM) as measured by a spectrofluorometer. FO-PSe was oxidized by 1 equiv. of added $O_2^{\bullet-}$, after which the solution was treated with 2 equiv. of GSH; (b) Fluorescence responses of FO-PTe (10.0 μM) to redox cycles. FO-PTe was oxidized by 1 equiv of added $O_2^{\bullet-}$ the solution was treated with 2 equiv of GSH. When the fluorescence returned to the baseline level, another 1 equiv of $O_2^{\bullet-}$ was added to the mixture.

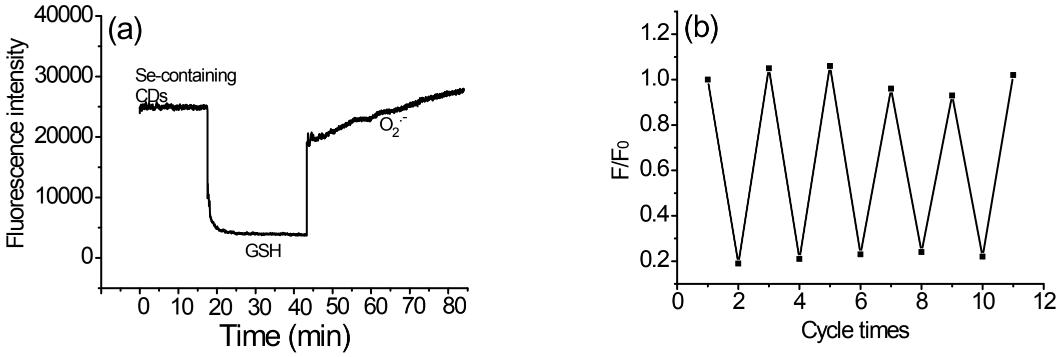

Fig. S9 (a) Time course of Se-containing CDs (10.0 μg/mL) as measured by a spectrofluorometer. Se-containing CDs were reduced by 1 equiv of added GSH, after which the solution was treated with 2 equiv of $O_2^{\bullet-}$; (b) Fluorescence redox cycles responses of Se-containing CDs. The Se-containing CDs were reduced by 1 equiv of added GSH the solution was treated with 2 equiv of $O_2^{\bullet-}$. When the fluorescence returned to the baseline level, another 2 equiv of $O_2^{\bullet-}$ was added to the mixture.

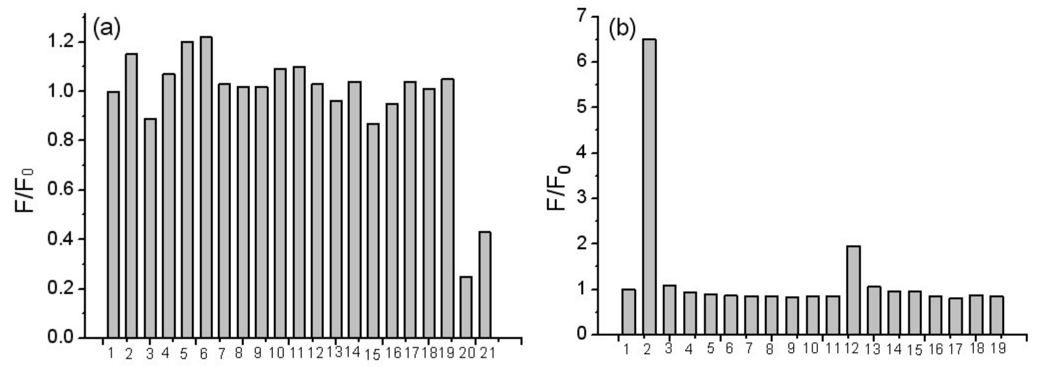

Fig. S10 Fluorescence responses of Se-containing CDs (a: SeO-CDs, b: Se-CDs, respectively) to various analytes. (a) 1, Se-containing CDs, 10 μg/ml; 20, GSH, 0.1 mM; 21, Vc, 0.1mM; (b) 1, Se-containing CDs + GSH; 2-19 in Fig. (a) and Fig. (b) was the same analytes, but different in concentration: 2, KO_2 (c: 0.05 mM, d: 0.01 mM); 3, ·OH, 0.05 mM; 4, ButooH, 0.05 mM; 5, $^1O_2$, 0.05 mM; 6, H_2O_2, 0.05 mM; 7, HClO, 0.05 mM; 8.K^+, 20 mM; 9, Na^+, 20 mM; 10, Ca^{2+}, 2 mM; 11, Mg^{2+}, 2 mM; 12, Cu^{2+}, 0.2 mM; 13, Cu^+, 0.2 mM; 14, Fe^{2+}, 0.2 mM; 15, Fe^{3+}, 0.2 mM; 16, Zn^{2+}, 2 mM; 17, Ni^{2+}, 0.2 mM; 18, Al^{3+}, 0.2 mM; 19, Mn^{2+}, 0.2 mM.

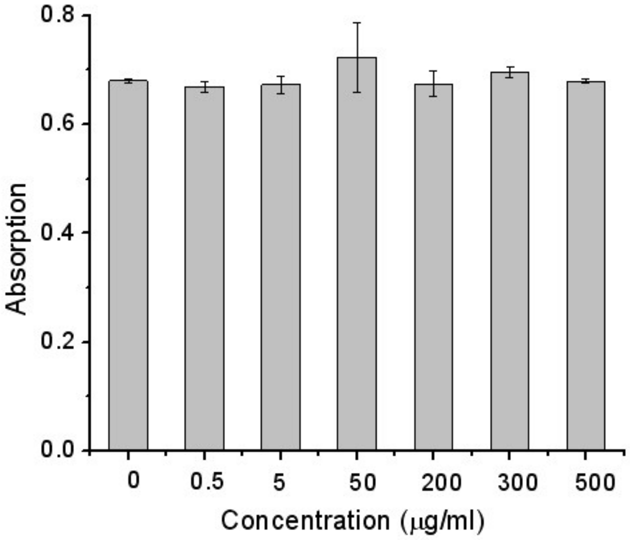

Fig. S11 Viability of Hepatocyte cells in the presence of Se-CDs as measured by using MTT assay. The cells were incubated with probe for 12 h.

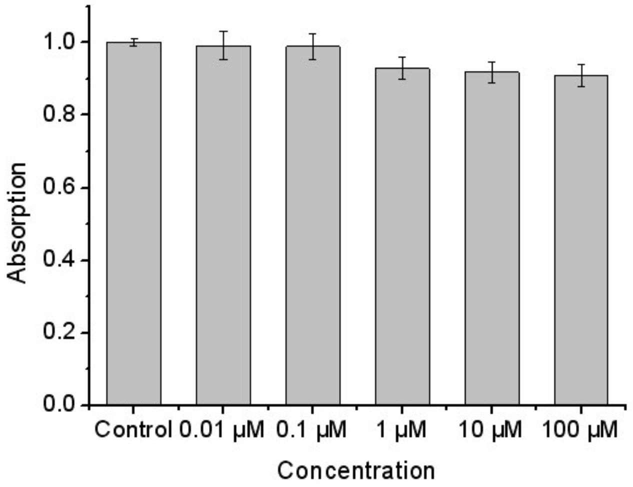

Fig. S12 Viability of Hepatocyte cells in the presence of FO-PTe as measured by using MTT assay. The cells were incubated with probe for 12 h.

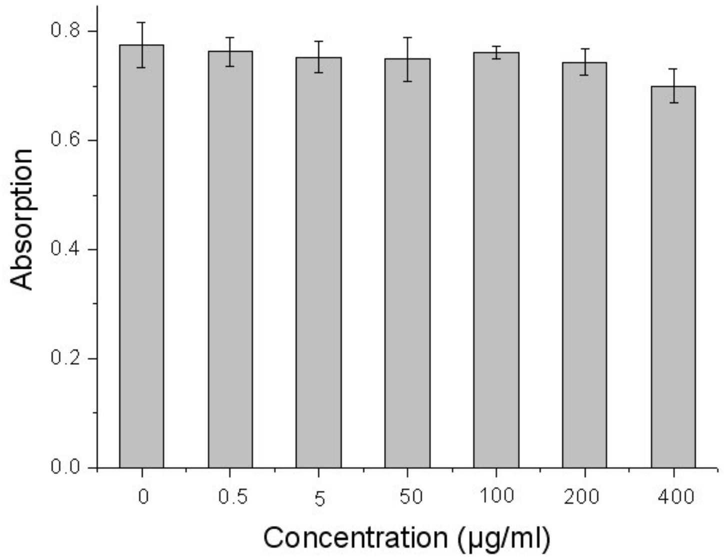

Fig. S13 Viability of Hepatocyte cells in the presence of Te-CDs as measured by using MTT assay. The cells were incubated with probe for 12 h.

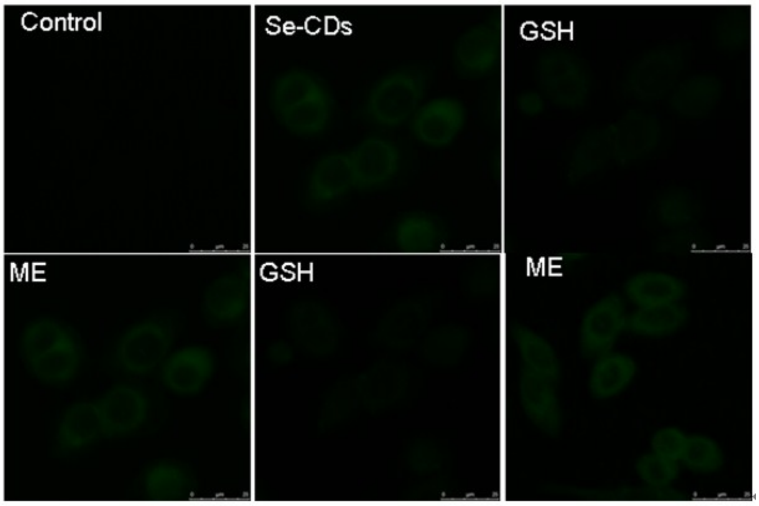

Fig. S14 Fluorescence imaging of reversible cycles of Se-CDs (10.0 μg/ml) in living mice macrophages (RAW264.7). (a) Fluorescence images of the CDs-loaded cells with GSH, and stimulated with 2-Methoxyestradiol (ME). The scale bar is 25 μm. Two-photon images were acquired using 800 nm excitation and two-photon fluorescent emission windows: 500-600 nm.

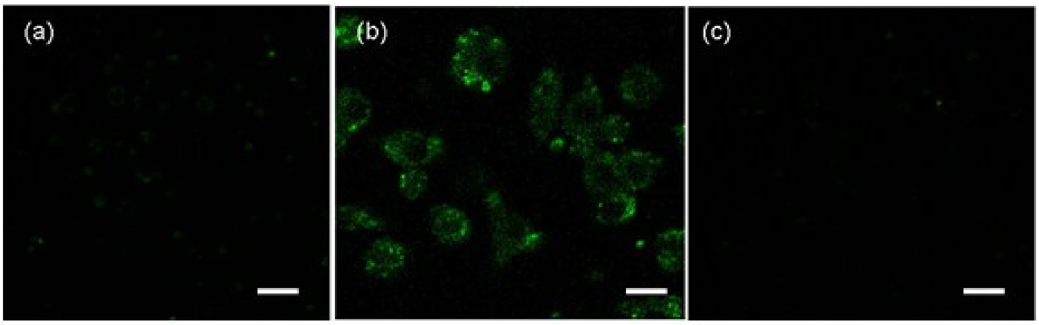

Fig. S15 Fluorescence images of $O_2^{\bullet-}$ in living mice macrophages (RAW264.7) under different conditions with probe FO-PTe. Mice macrophages were incubated with 10 μM probe. (a) Fluorescence image of the probe in cells. (b) Fluorescence image upon stimulation with PMA. (c) Fluorescence image upon stimulation with GSH. The scale bar is 15 μm. Two-photon images were acquired using 800 nm excitation and two-photon fluorescent emission windows: 400-500 nm.

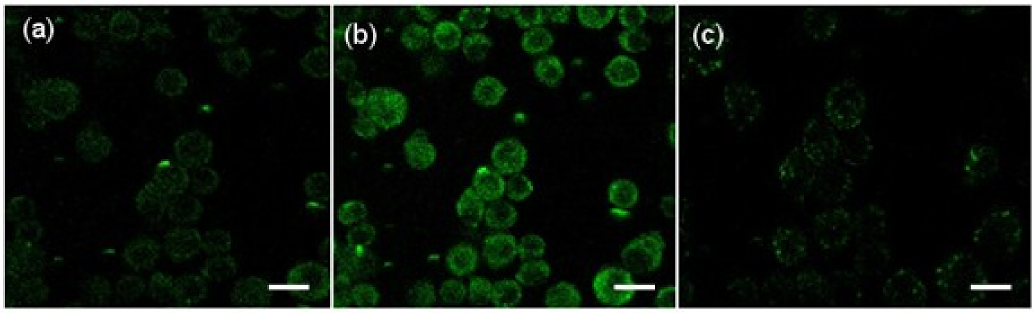

Fig. S16 Two-photon fluorescence images of living mice macrophages (RAW264.7) under different conditions with Te-CDs (5.0 μg/ml). (a) CDs-loaded fluorescence image. (b) Fluorescence image upon stimulation with PMA. (c) Fluorescence image upon stimulation with GSH in the stimulated cells. The scale bar is 15 μm. Two-photon images were acquired using 800 nm excitation and two-photon fluorescent emission windows: 400-500 nm.

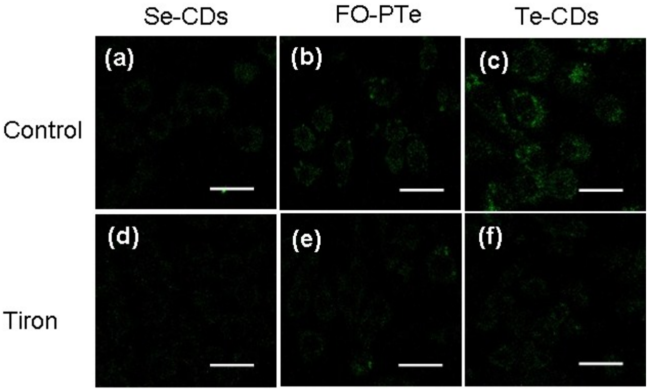

Fig. S17 Two-photon fluorescence images of $O_2^{\bullet-}$ levels in macrophages (RAW264.7). (a), (b), (c) fluorescence imaging of $O_2^{\bullet-}$ by Se-CDs, FO-PTe, and Te-CDs, respectively; (d), (e), (f) imaging of $O_2^{\bullet-}$ in macrophages were incubated with 10 μM Tiron for 30 min, then incubated by Se-CDs, FO-PTe, and Te-CDs, respectively. The concentration of the Te-CDs Se-CDs, FO-PTe was 5.0 μg/ml, 10.0 μg/ml, 10.0 μM, respectively. The scale bar is 20 μm. Two-photon images were acquired using 800 nm excitation and two-photon fluorescent emission windows: 400-500 nm for FO-PTe and Te-CDs, respectively. 500-600 nm for Se-CDs.

.

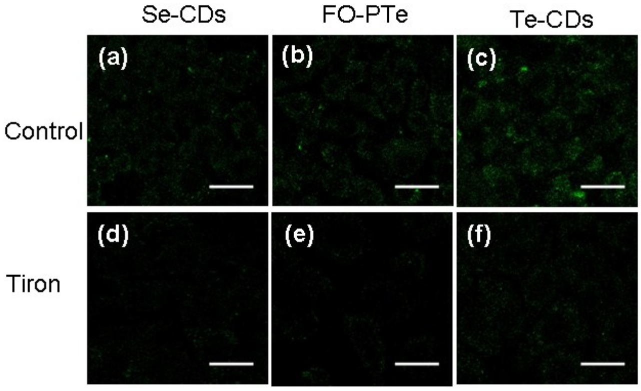

Fig. S18 Two-photon fluorescence images of $O_2^{\bullet-}$ levels in HepG2 cells. (a), (b), (c) imaging of $O_2^{\bullet-}$ by Se-CDs, FO-PTe, and Te-CDs, respectively; (d), (e), (f) imaging of $O_2^{\bullet-}$ in HepG2 cells were incubated with 10 μM Tiron for 30 min by Se-CDs, FO-PTe, and Te-CDs, respectively. The concentration of the Te-CDs Se-CDs, FO-PTe was 5.0 μg/ml, 10.0 μg/ml, 10.0 μM, respectively. The scale bar is 20 μm. Two-photon images were acquired using 800 nm excitation and two-photon fluorescent emission windows: 400-500 nm for FO-PTe and Te-CDs, respectively. 500-600 nm for Se-CDs.

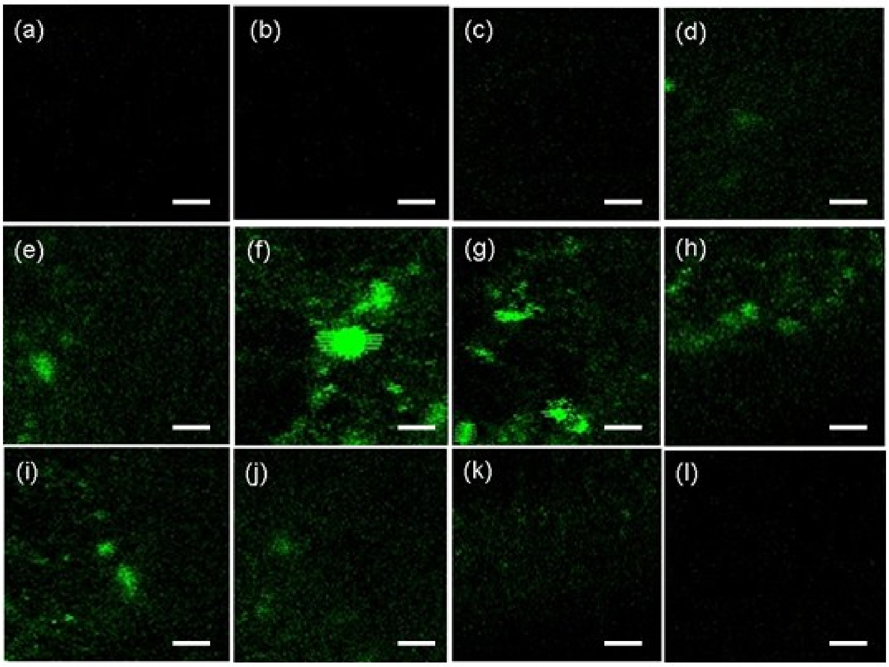

Fig. S19 The changes of $O_2^{\bullet-}$ levels at different depth in the abdomen of mice with Te-CDs (5.0 μg/ml). Images were acquired using 800 nm two-photon excitation (Fig. a-l was 69 μm, 130 μm, 194 μm, 236 μm, 330 μm, 412 μm, 525 μm, 598 μm, 633 μm, 782 μm, 836 μm, 871 μm, respectively). The scale bar is 150 μm. Two-photon fluorescent emission windows: 400-500 nm.

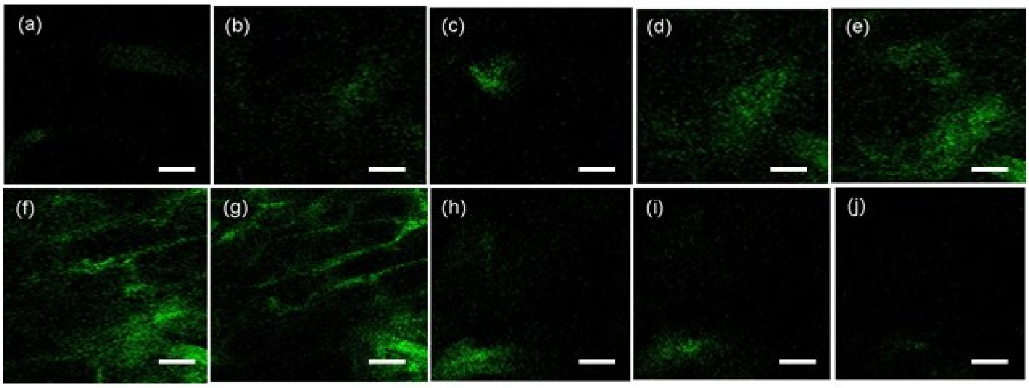

Fig. S20 The changes of $O_2^{\bullet-}$ levels at different depth in the abdomen of mice with FO-PTe (10.0 μM). Images were acquired using 800 nm two-photon excitation (Fig. a-j was 42 μm, 64 μm, 98 μm, 152 μm, 198 μm, 278 μm, 366 μm, 442 μm, 492 μm, 568 μm, respectively). The scale bar is 150 μm. Two-photon fluorescent emission windows: 400-500 nm.

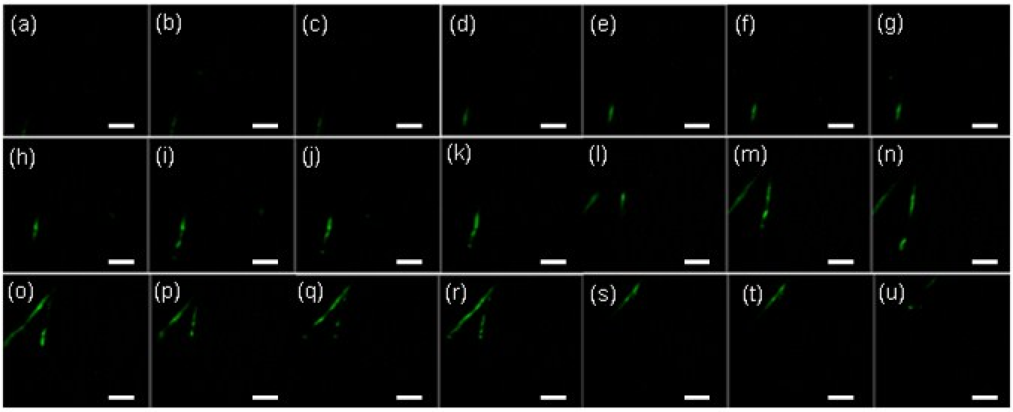

Fig. S21 The changes of $O_2^{\bullet-}$ levels at different depth in the abdomen of mice with Se-CDs (10.0 μg/ml). Images were acquired using 800 nm two-photon excitation (Fig. a-u was about 15 μm, 28 μm, 41 μm, 54 μm, 67 μm, 80 μm, 93 μm, 106 μm, 119 μm, 132 μm, 145 μm, 158 μm, 171 μm, 184 μm, 197 μm, 210 μm, 223 μm, 236 μm, 249 μm, 262 μm, 275 μm, respectively). The scale bar is 120 μm. Two-photon images were acquired using 800 nm excitation and two-photon fluorescent emission windows: 500-600 nm.

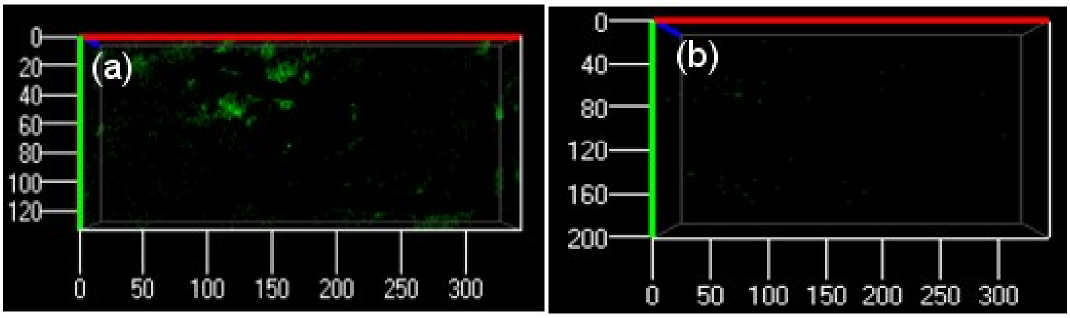

Fig. S22 Two-photon fluorescence images of $O_2^{\bullet-}$ levels in the abdomen of the depression mice with Te-CDs. (a) The 3D distribution of $O_2^{\bullet-}$ by Te-CDs; (b) The 3D distribution of $O_2^{\bullet-}$ in depression mice were incubated with 10 μM Tiron for 30 min. The concentration of the Te-CDs was 5.0 μg/ml. The images were acquired using 800 nm two-photon excitation, and two-photon fluorescent emission windows: 400-500 nm.
